# Hipoxia modulates the secretion of growth factors of human umbilical cord-derived mesenchymal stem cells

**DOI:** 10.37796/2211-8039.1416

**Published:** 2023-09-01

**Authors:** Arfianti Arfianti, Ulfah, Leopold S. Hutabarat, G Agnes Ivana, Anisa D. Budiarti, Nabilla S. Sahara, Nicko P.K. Saputra

**Affiliations:** a Department of Medical Biology, Faculty of Medicine, Universitas Riau, Pekanbaru, 28133, Indonesia; b Department of Anatomy, Faculty of Medicine, Universitas Riau, Pekanbaru, 28133, Indonesia; c Undergraduate Program, Faculty of Medicine, Universitas Riau, Pekanbaru, 28133, Indonesia; d LONTAR Laboratory, Faculty of Medicine, Universitas Riau, Pekanbaru, 28133, Indonesia; e Department of Obstetrics and Gynecology, Faculty of Medicine, Universitas Riau, Pekanbaru, 28133, Indonesia

**Keywords:** CoCl_2_, Hepatocyte growth factor, Hypoxia, Mesenchymal stem cell, Vascular endothelial growth factor

## Abstract

**Background:**

Mesenchymal stem cell (MSC) has great potential as therapies due its ability to regenerate tissue damage and promote tissue homeostasis. Preconditioning of MSC in low oxygen concentration has been shown to affect the therapeutic potential of these cells. This study aimed to compare the characteristic and secretion of trophic factors of MSCs cultured under hypoxia and normoxia.

**Methods:**

MSCs were isolated from Wharton’s jelly of human umbilical cord (UC) tissue by explant method and characterized by flow cytometry. Following 24 h of CoCl_2_-induced hypoxic culture, the viability and metabolic activity of MSC were analyzed by trypan blue exclusion test and methyl thiazolyl tetrazolium (MTT) assay, respectively. The secretion of hepatocyte growth factor (HGF) and vascular endothelial growth factor (VEGF) was assessed in conditioned medium using enzyme-linked immunosorbent assay (ELISA) method.

**Results:**

Flow cytometry analysis showed >99% of the population of MSCs cells were positive for CD73 and CD90 and > 62% were positive for CD105. While the cell viability of MSC was not affected by hypoxic cultured condition, the metabolic activity rate of these cells was decreased under hypoxic conditioning. In line with reduced metabolic activity, hypoxic human UC-derived MSC produced less HGF than normoxic counterpart. Compared to normoxic MSC, hypoxic preconditioned MSC secreted higher level of VEGF in the conditioned medium (p < 0.05).

**Conclusions:**

Hypoxia decreased the metabolic activity of MSCs associated with the modulation of HGF and VEGF secretions. It is suggested that hypoxia may also affect the therapeutic capacity of MSC cells.

## 1. Introduction

Mesenchymal stem cells (MSCs) are multi-potent progenitor cells capable of self-renewing and differentiating into osteoblast, chondrocytes, adipocytes or other connective tissues. Recently, MSC has gained significant interest as a potential candidate for cell-based therapy due to their immunomodulatory and regenerative properties [1,2]. Growing evidence from pre-clinical studies have demonstrated the therapeutic effect of MSC on neurodegenerative and inflammatory diseases. Likewise, there are more than 1000 clinical trials exploring MSC-based therapy that have been conducted against various conditions including graft-versus-host-disease (GvHD), osteoarthritis, multiple ischemic heart disease, cardiovascular disease, liver cirrhosis and corona virus disease-19 (Covid-19) [1,3].

Mesenchymal stem cell can be isolated from any tissues including bone marrow, adipose, Wharton’s jelly, umbilical vein endothelial cells and the placenta [1]. Although bone marrow-derived MSC is the most extensively studied, human umbilical cord (UC) is an attractive source for MSCs due its non-invasive sample collection, robust proliferative capacity and high immunomodulatory activity [4]. Human UC-derived MSC is a potential autologous or allogenic agent for treating diabetes and its complications, liver disease, systemic lupus erythematosus, arthritis, cerebrovascular diseases, heart diseases, spinal cord injury, respiratory diseases, viral infections, and other diseases [5]. Despite this attractive therapeutic strategy, more studies are needed to improve the understanding of mechanisms of action, cell stability and optimal culture condition to achieve significant therapeutic effects.

The therapeutic properties of MSCs are mediated not only through cell–cell contact but also via secretome, which is a complex mixture of soluble products secreted by MSCs. Secretome contains growth factors (including hepatocyte growth factor [HGF] and vascular endothelial growth factor [VEGF]), cytokines, microvesicles and exosomes and are considered as active pharmaceutical components that have potential therapeutic effects on various diseases with organ damage and impaired function [6,7]. Mesenchymal stem cells are able to replace cell damage, modulate inflammatory process and modulate cell function [8]. In addition, immunomodulatory capacity of MSC has been associated with the secretion of transforming growth factor beta (TGF-β) and IL-10 [6]. TGF-β plays important role in the regulation of immune response through the suppression of inflammatory response. Furthermore, TGF-β-derived MSC promotes macrophage M2-like polarization leading to reducing inflammatory reaction [9].

Despite well-documented of preclinical data and increasing number of clinical trials, MSC-based therapy still has not been implemented in clinical practice due to inability to meet primary efficacy end points [1,3]. Several factors may contribute to the failure of MSC to meet the clinical expectations, including heterogeneity of cell populations, inconsistency in cell stability, differentiation and immunomodulatory capacity [3]. Regular cultures expose MSCs with higher concentration of oxygen (normoxia) than *in vivo* concentration (hypoxia) [10]. Therefore, it is suggested that hypoxia preconditioning will be beneficial for improving therapeutic efficacy of MSC. To test this hypothesis, we compared the viability and proliferation rates of MSC cultured under hypoxia and normoxia conditions. We then examined whether hypoxia has any effects on the secretion of trophic factors by human UC-MSC.

## 2. Material and methods

### 2.1. Isolation and expansion of MSCs

Human umbilical cords were collected during elective cesarean section of women fulfilling the inclusion criteria: age 20–35 years, not *in partu*, negative for hepatitis B, HIV and SARS-CoV2 infection. The exclusion criteria are women with comorbidities including diabetes, hypertension in pregnancy, pre- and post-partum bleeding. Written informed consent was obtained from all participants and the study has been approved by the Research Ethics Committee of Faculty of Medicine Universitas Riau, Pekanbaru Indonesia (B/0477UN19.5.1.1.8/UEPKK/2021). Umbilical cord was stored in Dulbecco’s phosphate-buffered saline (DPBS) containing penicillin 100 U/mL, streptomycin 100 μg/mL (Gibco, Rockville, MD, USA) at 4 °C. MSCs were isolated from UC’s Wharton’s Jelly (WJ) using an explant protocol with slight modifications [11]. Blood vessels were removed from UC and the WJ was stripped away from the blood vessels and minced into small pieces. Explants were transferred to 75 cm^2^ culture flask and cultured in low glucose Dulbecco’s Modified Eagle Medium (DMEM; Gibco), 1% of penicillin-streptomycin and 10% fetal bovine serum (FBS; Sigma–Aldrich, Darmstadt, Germany) in a CO_2_ incubator (ESCO life science Bintan Indonesia) with humidified air containing 5% CO_2_ at 37 °C. Media were changed after 5 days of undisturbed incubation and replaced every fourth day up to 2 weeks. When cells reached ~80% confluence, the cell monolayer was detached using trypsin–EDTA (0.25%) and reseeded to a density of 10^4^ cells/cm^2^. Cell viability was examined using trypan blue exclusion test (Sigma–Aldrich, Darmstadt, Germany).

### 2.2. Characterization of MSCs

Morphology of MSC was assessed using inverted microscope (Olympus, Japan). Mesenchymal stem cell characterization was based on criteria defined by the International Society for Cellular Therapy [12]. The cultured MSCs at passage 3 were immunophenotyped using BD Stemflow™Human MSC Analysis kit (BD Biosciences). Cells were washed with DPBS, trypsinized and resuspended into 3 × 10^6^ cells/mL. Cells were stained with following antibodies CD105-Per-CP-Cy™5.5, CD90-fluorescein isothiocyanate (FITC), CD73-allophycocyanin (APC) and isotype-matched control IgG labelled with phycoerythrin (PE) was used as controls. Stained cells were evaluated with BD FACS CANTOII Flow Cytometer (BD Biosciences) with at least 10,000 events per sample, and the data were analyzed with BD FACSDiva™ software (BD Biosciences).

### 2.3. Induction of hypoxia in MSC culture

Hypoxia in MSC culture was induced by Cobalt (II) Chloride hexahydrate (CoCl_2_, Sigma–Aldrich, Darmstadt, Germany). Stock solution of 25 mM CoCl_2_ was prepared in sterile distilled water. MSCs were seeded at a density of 5 × 10^3^ cells per well in 96-well culture plates for MTT proliferation assay and 10^5^ cells per well on 6-well culture plate for analyses. After reaching ~80% confluency, culture media were replaced by adding new media containing 100 μM of CoCl_2_ and cells were incubated for 24 h. The cultured MSCs at passages 3–5 were used in all experiments.

### 2.4. MTT assay for metabolic activity

The metabolic activity of MSCs was measured using 3–4, 5-dimethylthiazole-2-yl)-2,5-diphenyltetrazolium bromide (MTT) assay. Cells were plated in 96-well culture plates at a density of 5 × 10^3^ cells per well and treated with CoCl_2_ to induce hypoxia or cultured at normal oxygen concentration (normoxia). After 24 h of incubation, the culture media were discarded and 200 μL of MTT reagent (2 mg7mL dissolved in DPBS) was added to each well. After incubation for 3 h at 37 °C, purple formazan precipitates were dissolved using an organic solvent of DMSO and absolute ethanol (1:1). The absorbance was read at a wavelength of 570 nm using Multiskan Sky microplate spectrophotometer (Thermo Scientific, USA). Metabolic activity was expressed as a percentage of absorbance relative to normoxic group.

### 2.5. Measurement of cytokines and growth factors in conditioned medium

Following 24 h of normoxia or hypoxia incubation, culture medium was collected and centrifuged at 1000×*g* for 10 min at 4 °C. Levels of IL-10, TGF-β1, VEGF and HGF in culture media of MSCs were measured using Elikine™ human IL-10, TGF-β1, VEGF and HGF ELISA kit (Abbkine, Wuhan, China), respectively according to manufacturer’s instructions.

### 2.6. Data analysis

Data were presented as mean ± standard error of the deviation (SD). Significant differences on the secreted levels of cytokines and growth factors as well as cell viability and metabolic activity were analyzed using independent t test. The statistical tests were performed using GraphPad Prism 7 for MacOS X and *p* value < 0.05 was deemed significant.

## 3. Results

### 3.1. Immunophenotypes and morphology of mesenchymal stem cells

**Fig. 1:**
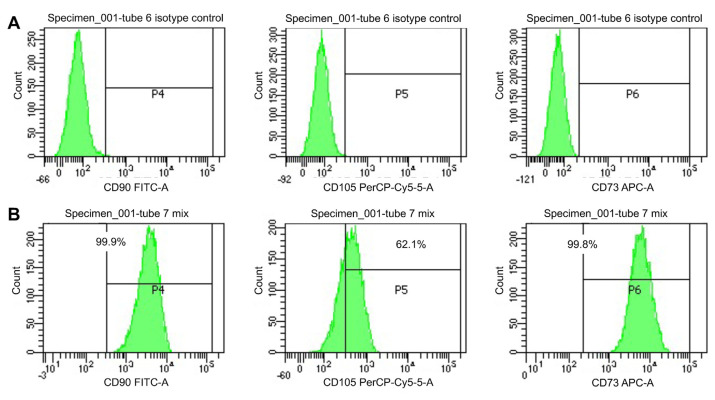
Immunophenotyping of human umbilical cord-derived mesenchymal stem cells 759460076200Mesenchymal stem cell culture at passage 3 was immunophenotyped using flow cytometry using BD Stemflow; Human MSC Analysis kit (BD Biosciences). A) Isotype controls for B) CD90, CD105 and CD73.

**Fig. 2:**
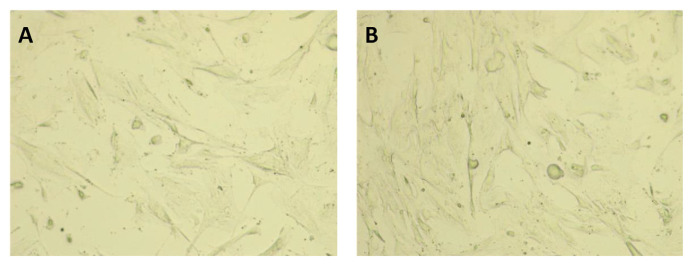
Morphology of mesenchymal stem cells under normoxic and hypoxic cultures Representative phase-contrast images of mesenchymal stem cells after 24 hours of A) normoxic and B) hypoxic conditions on human umbilical cord-derived mesenchymal stem cells.

The immunophenotyping of MSCs was carried out by flow-cytometry at passage 3 as shown in Fig. 1. Among the three surface markers for MSC, CD90 and CD73 were expressed in 99.9% and 99.8% of MSCs, respectively. On the other hand, CD105 were only detected in 62.1% of cells. Based on phase contrast microscopy analysis, the morphology of MSCs was not different between cells cultured under hypoxia and normoxia conditions; most UC-derived MSCs displayed fibroblast-like shape but few colonies of flattened cells were also detected (Fig. 2).

### 3.2. Effect of hypoxia on cell viability and metabolic activity

**Fig. 3:**
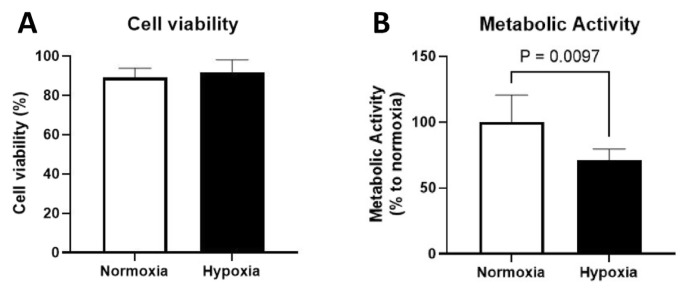
Effect of hypoxic culture on cell viability and metabolic activity of human umbilical cord-derived mesenchymal stem cells Hypoxia in human umbilical cord-derived mesenchymal stem cells was induced by CoCl_2_ treatment for 24 hours. (A) Cell viability was measured by trypan blue exclusion test and (B) metabolic activity was examined by MTT assay (n=6).

The cell viability was examined by trypan blue exclusion test (Fig. 3). The results showed that the cell viability was 93.3% under hypoxia and 88.9% under normoxia cultures; there was no significant loss of cell viability under hypoxia condition (p > 0.05). However, the metabolic activity rate of MSCs was markedly reduced as much as ~30% into 71.2% under hypoxia condition compared to those of normoxia culture (p = 0.0097).

### 3.3. Effect of hypoxia on the secretion of cytokines and growth factors

**Fig. 4:**
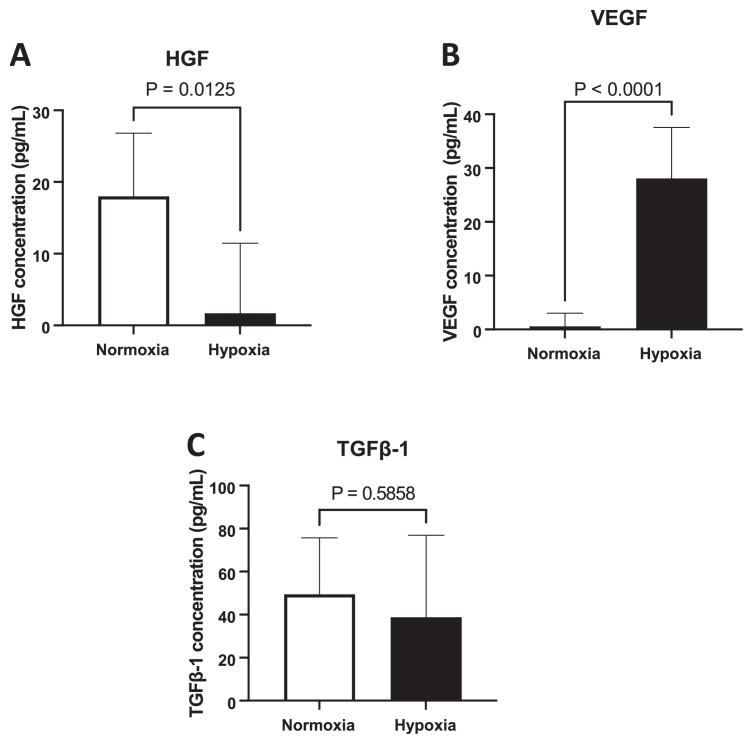
Effect of hypoxic culture on HGF, VEGF and TGF-1 secretion from human umbilical cord-derived mesenchymal stem cells Hypoxia in human umbilical cord-derived mesenchymal stem cells was induced by CoCl_2_ treatment for 24 hours. The secretion of (A) HGF, (B) VEGF and (C) TGF 1 in conditioned media was measured by enzyme-linked immunosorbent assay (ELISA) method

Modulation of the secretion of cytokines and growth factors by hypoxia was examined by measuring the levels of these biologic factors in conditioned medium by ELISA (Fig. 4). The levels of VEGF in hypoxic-conditioned medium (28.1 ± 9.5 pg7mL) increased nearly 30 times compared to normoxic-conditioned medium (0.6 ± 2.45) (p < 0.0001). In contrast, HGF secreted by MSCs was significantly reduced from 18 ± 8.8 pg/mLin normoxic culture into 1.7 ± 9.8 pg/mL in hypoxic culture (p = 0.0125). We also measured the concentrations of IL-10 in conditioned medium, but the levels were undetectable both in hypoxia and normoxia conditions. Further, the results revealed that the levels of TGF-β1 were 49.45 ± 26.3 pg/mL in normoxia culture and these did not significantly change following hypoxia culture (38.8 ± 38.1 pg/mL).

## 4. Discussion

In this study, we showed that CoCl_2_-induced hypoxia decreased the metabolic activity of human UC-derived MSCs and led to a significant modulation in the secretion of HGF and VEGF. We also demonstrated that CoCl_2_ treatment did not affect the cell viability and secretion of TGF-β1 in human UC-derived MSCs.

Mesenchymal stem cell (MSC) is gaining attention as a potential candidate for treating various tissue injuries due to its regenerative, immunomodulatory and multi-lineage differentiation capacities. However, safety issues regarding tumorigenicity, heterogeneity of cell populations, and poor survival over long-term expansion limit the clinical application of MSCs. To overcome these obstacles, scientists have shifted their attention to various biological factors secreted by MSCs containing cytokines, growth factors, angiogenic factors, hormones, chemokines, extracellular matrix proteins, extracellular matrix proteases, and genetic materials.

First, we confirmed the morphology of the human UC-derived MSCs which predominantly showed elongated, fibroblast-like cells. It is well-known that MSCs consist of a heterogeneous cell population morphologically and biologically. There are three distinct populations of MSCs, namely small starshaped cells, fibroblastic-like, spindle-shaped, and large, flattened cells. According to previous studies, these three subpopulations have different proliferative abilities, where small star-shaped cells have the highest cell proliferation activity, spindle-shaped cells have the highest ability to differentiate into cartilage tissue [13]. The International Society for Cellular Therapy (ISCT) has issued minimal criteria for identifying MSCs, including plastic-adherent in standard culture conditions, the expression of a certain panel of surface protein markers and multilineage differentiation [12,14]. In this study, MSCs exhibited >99% of cell surface markers specific for MSCs, including CD90 and CD73. This indicated that the isolated and expanded MSCs had high purity and possessed surface protein markers in accordance with the criteria by the ISCT [12]. However, the population of cells expressing the CD105 marker was quite low (62.1%) compared to the ISCT criteria, which was >95%. Previous studies have shown that MSCs with low CD105 expression may affect the ability of MSCs to secrete anti-inflammatory factors through the modulation of lymphocyte cell activation and pro-inflammatory cytokine production [15] Whether the low expression of CD105 may influence the therapeutic efficacy of MSCs is warrant further studies.

This study showed that hypoxia did not have any effect on cell viability as determined by trypan blue exclusion test but decreased the metabolic activity of human UC-MSCs as measured by MTT assay. The inhibitory effects of hypoxia on metabolic activity were shown in human MSC isolated from bone marrow which was associated with reduced levels of intracellular ATP. This was partly due to the metabolic shift towards a more glycolytic state, rather than oxidative phosphorylation [16]. In response to low concentration of oxygen, the expression of hypoxia inducible factor α (HIF-1α) is increased to promote differentiation and immunosupprecive capacity of MSC [17]. Likewise, hypoxia-induced metabolic reprogramming has been used to mitigate the metabolic shock experienced by MSCs after transplantation, leading to enhanced cell survival and retention [18,19].

We then demonstrated that VEGF levels in the hypoxia conditioning media of human UC-MSC were significantly increased compared to normoxic cells, suggesting hypoxia may stimulate the expression of VEGF within UC-MSC. Our data further support previous studies showing that hypoxia not only affects the VEGF expression in hUC-derived MSC [20] but also in various sources of MSC including adipose and bone marrow [21,22]. Vascular endothelial growth factor (VEGF) is a potent angiogenic factor that promotes neovascularization by stimulating vasculogenesis, angiogenesis and vascular permeability. Consequently, VEGF has been reported to contribute to various pathological conditions such as tumorigenesis, diabetic retinopathy, age-related macular degeneration arteriosclerosis and stroke [22]. In addition, VEGF play a critical role in the formation of vasculature during embryonic development as VEGF deficient embryos showed impaired blood vessels [23]. Further, co-culture experiment demonstrated that MSC was able to induce the differentiation of endothelial progenitor cells through a paracrine mechanism and VEGF was suggested to mediate the communication between these two cells [24].

This study showed that hypoxia condition reduced HGF secretion in human UC-MSC. In contrast, previous studies showed that hypoxia preconditioning increased HGF secretion in MSCs [25,26]and MSC transfected with HGF possess better antifibrotic vascular repair mesenchymal to epithelial transformation (MET), migratory capacity [27,28]. Hepatocyte growth factor is a pleiotropic growth factor promoting cell proliferation, motility, survival, and morphogenesis, and angiogenesis. Thus, reduced proliferation in hypoxic human UC-MSC under hypoxia conditioning was in line with the decreased levels of HGF in hypoxia conditioned media. Schive et al. also reported that hypoxia-treated MSC exhibited decreased levels of HGF compared to normoxia-conditioned MSC [29].

In this study, hypoxia culture did not change the secretion of TGF-β1 in human UC-MSC. In contrast, Hung et al. reported that hypoxia increased the secretion of TGF-β1 [30]. TGF-β signaling is important for regulating cell proliferation, differentiation, tissue repair and tumorigenesis. TGF-β induces the activation of fibrosis to produce extracellular matrix (ECM) and overexpression of TGF-β signaling promotes epithelial–mesenchymal transition (EMT) [31]. Thus, TGF-β is an essential cytokine in the pathogenesis of fibrosis and tumor progression. Indeed, Hung et al. reported that conditioned medium from hypoxia-treated bone marrow-derived MSC was able to promote the proliferation of breast cancer cell lines through the induction of TGF-β1 secretion [30]. Interestingly, the expression of components of TGF-β signaling and HIF-1α were associated with a poor prognosis of patients with clear cell renal cell carcinoma. Further, HIF-1α enhanced the function of TGF-β1 to increase tumor cell glycolysis in hypoxia condition [32]. These data suggest that the nature of hypoxia within tumor microenvironment may synergize with TGF-β signaling to promote fibrosis and cancer development. Therefore, the implication of hypoxia preconditioning of MSC on the function of secreted TGF-β1 is needed to be confirmed before the application of MSC for therapeutic purposes.

With regard to the secretion of IL-10, we were not able to detect a measurable level of IL-10 in human UC-MSC under both hypoxia and normoxia condition. IL-10 is an anti-inflammatory cytokine which has the ability to modulate the inflammatory immune response by inhibiting T cell activation and cytokine production. It seems that IL-10 is not abundantly expressed in MSCs and hypoxia preconditioning is insufficient to increase IL-10 production in MSCs, at least not after 24 h of hypoxia culture as shown in this study. Other preconditioning approaches including cytokine stimulation, genetic modification or 3-dimensional culture may be required to enhance the expression of IL-10 within the MSCs and subsequently result in robust secretion of IL-10 [33].

This study, however, subjects to several possible limitations. First, we did not measure the O_2_ concentration within the cell culture and therefore not able to ensure whether the hypoxia condition was occurred in the cells. However, CoCl_2_ is known as a hypoxia-mimetic agent [34] and previous studies have demonstrated that CoCl_2_ treatment upregulated the expression of HIF-1α, a well-known cellular marker for hypoxia [35]. We also did not perform any functional assays to confirm whether the modulation of HGF and VEGF secretion in human UC-MSC by hypoxia has any implication on the therapeutic function of MSC. Therefore, further studies are required to confirm these findings.

In conclusion, the results of the present study demonstrated that short-term hypoxia enhanced the secretion of VEGF but reduced the production of HGF in human UC-derived MSCs without compromising the cellular viability. Hypoxia also decreased metabolic activity of MSC and it is of interest whether this metabolic shift would have an implication on the therapeutic effects of MSC including immunomodulatory, differentiation and regenerative capacities.
